# Emergence of Genotype 2.3.1 Multidrug-Resistant *Salmonella enterica* Serovar Typhi Strain, West Africa, 2021–2025

**DOI:** 10.3201/eid3210.260813

**Published:** 2026-10

**Authors:** Elisabeth Njamkepo, Maria Pardos de la Gandara, Carolina Silva Nodari, Alexandra Moura, François-Xavier Weill

**Affiliations:** Institut Pasteur, Université Paris Cité, Paris, France

**Keywords:** Salmonella enterica, bacteria, bacterial infection, Typhi, typhoid fever, genomic surveillance, antimicrobial resistance, West Africa, Senegal

## Abstract

Since 2021, a genotype 2.3.1, ciprofloxacin-resistant, multidrug-resistant (MDR) *Salmonella enterica* serovar Typhi strain increasingly has been detected in persons returning from West Africa. This strain locally acquired 2 different MDR plasmids over a ≈25-year period, underwent chromosomal integration of the IncHI1 plasmid MDR region, and finally acquired a *gyrA* mutation.

*Salmonella enterica* serovar Typhi is a human-restricted pathogen causing typhoid fever through fecally contaminated food or water (*1*). In 2021, an estimated 7.1 million typhoid fever cases caused 93,300 deaths worldwide (*2*). Sporadic antimicrobial resistance (AMR) was first detected in *Salmonella* Typhi in the 1950s, followed by epidemic multidrug-resistant (MDR) populations in the Americas and South/Southeast Asia during the 1970s–1980s. Those populations initially displayed resistance to chloramphenicol, streptomycin, tetracycline, and sulfonamides and then became resistant to ampicillin and cotrimoxazole, after the acquisition of large IncHI1 plasmids (*3*–*7*). One such MDR population, H58 (*3*), now genotype 4.3.1 (*8*), emerged in South Asia in the late 1980s and spread across Southeast Asia, Oceania, and East Africa (*9*,*10*). This H58 lineage first acquired an IncHI1 MDR plasmid (belonging to plasmid multilocus sequence type [pST] 6), followed by, during the 1990s, a point mutation in the chromosomal DNA gyrase gene, *gyrA* (often *gyrA*_S83F), which confers resistance to nalidixic acid (NAL^R^) and has decreased susceptibility to ciprofloxacin (now categorized as resistant to ciprofloxacin [CIP^R^]) according to established guidelines (Appendix 1). The MDR region initially located on the IncHI1 plasmid became stably integrated into the bacterial chromosome over time in lineage H58, and the IncHI1 plasmid was lost (*9*). This lineage subsequently became extensively drug-resistant after the acquisition of a new IncY plasmid carrying the extended-spectrum β-lactamase *bla*_CTX-M-15_ gene in Pakistan in 2016 (*11*).

In the 2000s, MDR *Salmonella* Typhi strains of non–4.3.1 genotypes emerged in West and Central Africa. In West Africa and neighboring Cameroon, the MDR genotypes were 3.1.1 (corresponding to haplotypes H56 and H42 [*3*] or cluster A [*12*]) and 2.3.1 (corresponding to H77 [*3*] or cluster C [*12*]). Both genotypes had predominantly acquired MDR plasmids of incompatibility group IncHI1 (pST2 with *dfrA15* as the class 1 integron gene cassette for 3.1.1 isolates and pST2 with *dfrA1-aadA1* for 2.3.1 isolates) (*12*,*13*). IncY plasmids in genotype 3.1.1 isolates and IncN (pST3) plasmids in 2.3.1 isolates were less frequent (*13*,*14*). Quinolone resistance was initially rare in these MDR genotypes; only 6 (4.7%) NAL^R^ isolates (all genotype 3.1.1) were found among 128 *Salmonella* Typhi isolates that were collected in Nigeria during 2008–2013 (*13*). However, a recent study showed that the proportion of genotype 3.1.1 NAL^R^ isolates reached >75% in Nigeria after 2015 (*15*). A similar trend has not been documented for genotype 2.3.1.

In France, enteric fevers have been notifiable diseases since 1903. In addition to mandatory reporting, clinical laboratories forward the isolates to the National Reference Center for *Escherichia coli*, *Shigella*, and *Salmonella* at the Institut Pasteur (Paris, France) on a voluntary basis. Most *Salmonella* Typhi isolates are obtained from travelers or immigrants, most of whom were infected in Asia and Africa. During 2016–2025, the center received 1,603 *Salmonella* Typhi isolates (1 per patient) for expert analysis (Appendix 1), including 167 of genotype 2.3.1. The median age for those 167 patients was 28 years (range 2–85 years); 64.7% were women and girls and 35.3% men and boys, and 135 (80.8%) had reported traveling to Africa.

We used conventional microbiologic examination and whole-genome sequencing to characterize an emerging MDR-CIP^R^ *S.* Typhi strain of genotype 2.3.1 isolated from travelers returning to France from West Africa. We also reconstructed its evolutionary history.

## The Study

In 2022, we obtained 35 MDR-CIP^R^ *Salmonella* Typhi isolates from persons returning from Senegal (Figure 1). All isolates belonged to genotype 2.3.1 and contained AMR genes *bla*_TEM-1_, *strA*, *strB*, *aadA1*, *sul1*, *sul2*, *dfrA1*, and *tet(B)* (this particular combination of AMR genes was thereafter named MDR profile A [MDRA]) and the point mutation *gyrA_*S83F, thus explaining their AMR phenotype (resistance to ampicillin, streptomycin, sulfonamides, trimethoprim, cotrimoxazole, tetracycline, nalidixic acid and ciprofloxacin [MIC range 0.125–0.25 mg/L]) (Appendix 2). Only 1 similar isolate had previously been documented in the Institut Pasteur registry, obtained from a case linked to Senegal in 2021 (Figure 1). The number of genotype 2.3.1 isolates linked to Senegal decreased from 21 in 2023 to 8 in 2024 but then peaked at 50 in 2025 (Figure 1). All 79 isolates recovered since 2023 were CIP^R^; 71 also displayed the MDRA profile (Appendix 2). We also identified in our database 52 additional genotype 2.3.1 genomes, obtained since 2022; of those, 39 carry the MDRA-CIP^R^ profile (37 also carry the *gyrA_*S83F mutation), and 11 originated from persons who had traveled to countries in West Africa (Guinea, Guinea-Bissau, Niger, Nigeria, Mauritania, The Gambia, and Benin) (Appendix 2).

**Figure 1:**
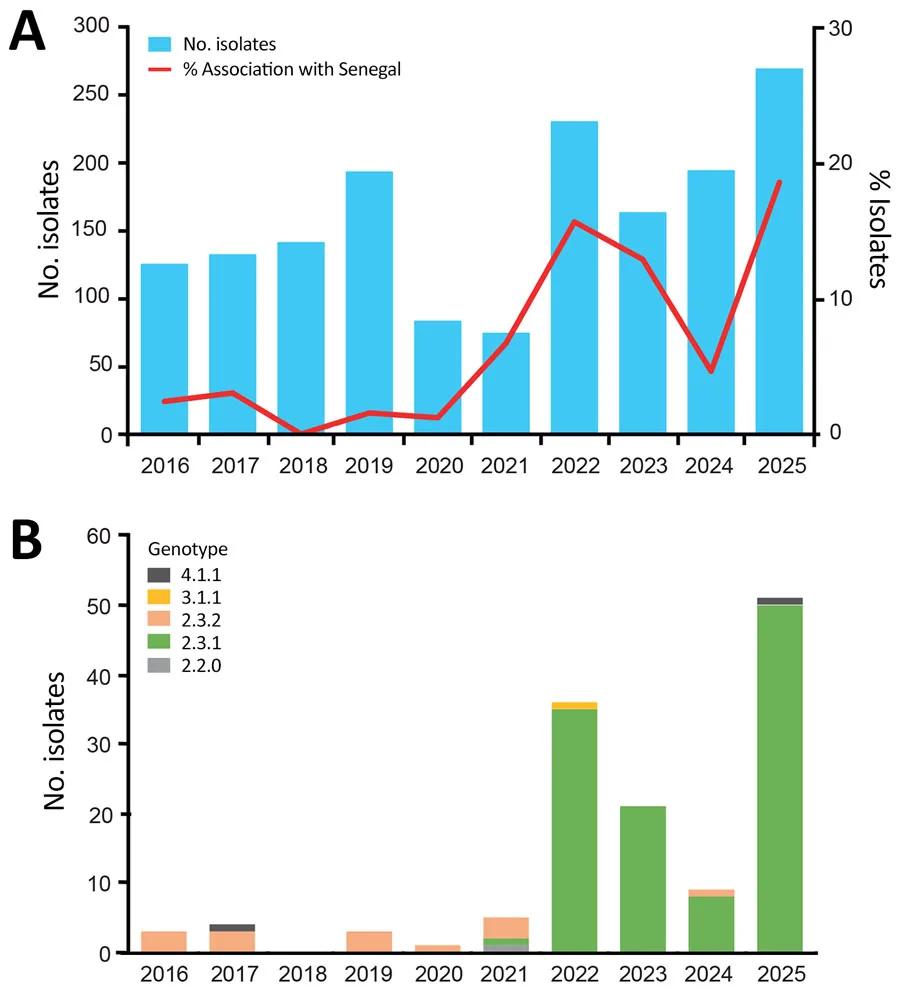
Distribution and genotype of *Salmonella enterica* serovar Typhi isolates in persons returning to France from Senegal in study of emergence of genotype 2.3.1 multidrug-resistant *Salmonella* Typhi strain, West Africa, 2016–2025. A) Total number of nonredundant *Salmonella* Typhi isolates received each year by France’s National Reference Center for *Escherichia coli*, *Shigella*, and *Salmonella* at the Institut Pasteur and percentage of those associated with Senegal. The unusually small number of *Salmonella* Typhi isolates received in 2020 and 2021 (<100 isolates) occurred in the context of COVID-19–related travel restrictions. B) Annual distribution of genotypes for *Salmonella* Typhi isolates obtained from persons returning from Senegal.

We constructed a maximum-likelihood phylogeny of 207 genomes of genotype 2.3.1 (167 from our study and 40 published genomes [*15*]), including the oldest strain, isolated in Cameroon in 1958 (Appendix 2) from 1,130 single-nucleotide variants. We observed 2 main types of MDR genomes differing in AMR gene content in the phylogenetic tree: MDRA (n = 161) and a second type of MDR genomes, which we named MDRB (n = 11), carrying *bla*_TEM-1_–like, *strA*, *strB*, *sul2*, *dfrA14*, and *tet(A)*. Most of the MDRB genomes clustered in the upper clades of the tree and contained a pST3 IncN plasmid (Figure 2). The MDRA genomes were located in the central and lower clades of the tree. Those in the central part contained the pST2 IncHI1 plasmid, and the oldest MDRA strains carrying that plasmid were from Cameroon in 2004. All 154 CIP^R^ isolates with the *gyrA*_S83F mutation (collected during 2021–2025 and belonging to the emerging MDR strain) clustered tightly together at the bottom of the tree (Figure 2), of which 144 had the MDRA profile. One published MDRA-CIP^R^ (*gyrA*_S83F) genome from a strain collected in Nigeria in 2019 (National Center for Biotechnology Information Sequence Read Archive [https://www.ncbi.nlm.nih.gov/sra] accession no. SRR10198914) appeared to be the nearest ancestor of this cluster (Figure 2; Appendix 2). 

**Figure 2:**
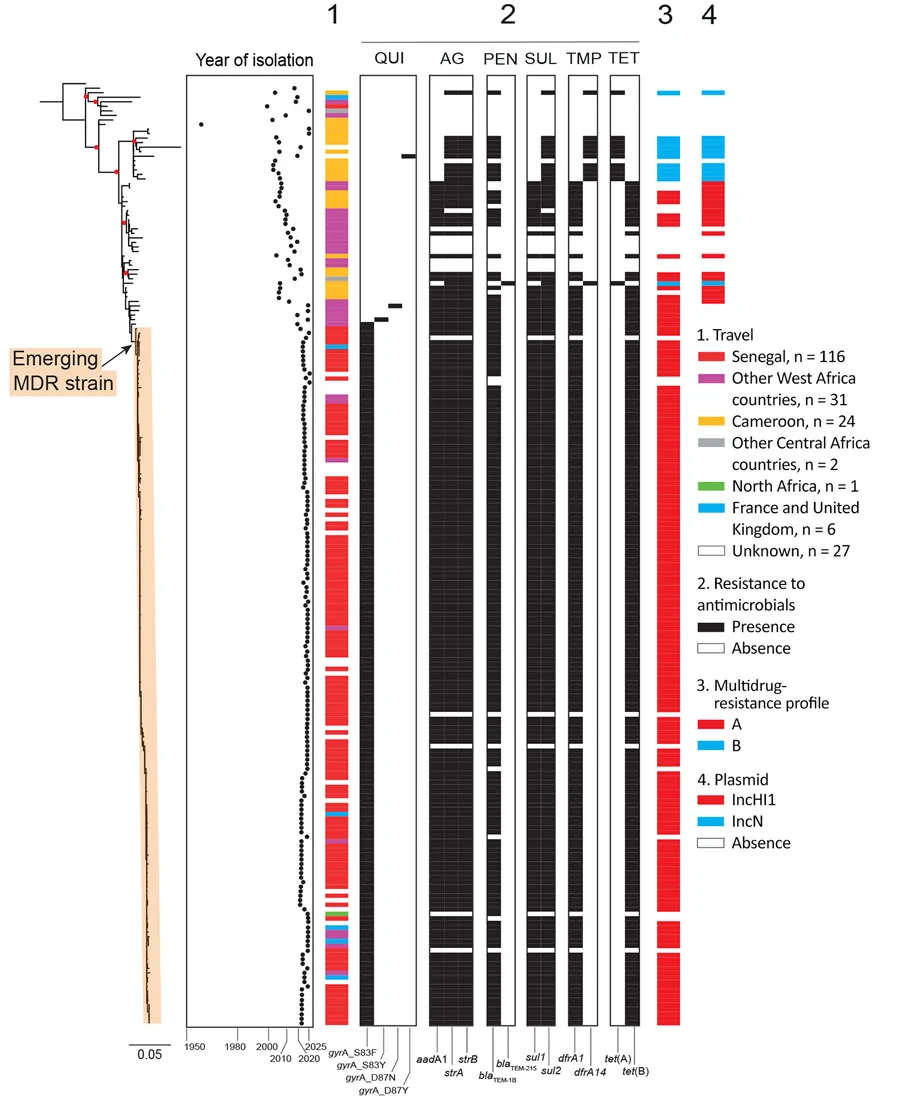
Maximum-likelihood phylogeny of 208 *Salmonella enterica* serovar Typhi isolates in study of emergence of genotype 2.3.1 MDR *Salmonella* Typhi strain, West Africa, 2021–2025. Included are 207 genotype 2.3.1 isolates (167 from France’s National Reference Center for *Escherichia coli*, *Shigella*, and *Salmonella* and 40 published genomes) and the reference genome CT18. CT18 (genotype 3.2.1) was used as the outgroup of the tree (its associated characteristics in terms of geographic origin, antimicrobial-resistance genes, and plasmid types are not indicated). Red color on dendrogram indicates nodes with bootstrap support >98%. Light orange rectangle indicates emerging MDR strain. Year of isolation is indicated, together with the travel information (when available) for the isolate, to the right of the tree, because those parameters serve as a proxy for the probable origin of the isolates. Resistance to certain antimicrobial drugs and the presence (black) or absence (white) of antimicrobial-resistance genes (names given along bottom of tree) also are indicated to the right of the tree. The MDR profiles are indicated in red (for profile A; *bla*_TEM-1_, *strA*, *strB*, *aadA1*, *sul1*, *sul2*, *dfrA1*, and *tet(B)*) or blue (for profile B; *bla*_TEM-1_–like, *strA*, *strB*, *sul2*, *dfrA14*, and *tet(A)*). The presence of plasmid markers is indicated in blue (for IncN) or red (for IncHI1). Scale bar indicates number of nucleotide substitutions per variable site. Information displayed in this figure can be found for each individual strain in Appendix 2 (https://wwwnc.cdc.gov/EID/article/32/10/26-0813-App2.xlsx). AG, aminoglycosides; MDR, multidrug-resistant; PEN, penicillins; QUI, quinolones; SUL, sulfonamides; TET, tetracyclines; TMP, trimethoprim.

We identified no plasmid in our emerging MDR strain, suggesting that the MDR region of the IncHI1 plasmid had been stably integrated into the chromosome. Long-read sequencing of 2 MDRA-CIP^R^ isolates (202210167 and 202207569) from the emerging MDR strain confirmed that hypothesis, revealing the integration of a 34,664-bp AMR region in the vicinity of chromosomal genes STY2890 and STY2889 (according to reference genome CT18) (Figure 3, panel A). That IS*26*-flanked AMR region contained all 8 AMR genes seen in MDRA isolates. In addition, long-read sequencing of MDRA isolates IPCP (Cameroon, 2006) and 201607514 (Chad, 2021) showed both contained a conjugative pST2 IncHI1 plasmid (188,859-bp pIPCP-1 in IPCP and 187,575-bp p201607514–2 in 201607514) (Appendix 1), indicating that the chromosome-borne AMR region originated from the pST2 IncHI1 plasmid. The region between nucleotides 2824 and 33844 of the AMR region integrated into the chromosome of the epidemic strain (Figure 3, panel B) was 100% identical to region 53376–84396 of the pIPCP-1 plasmid. The *bla*_TEM-1_ β-lactamase gene (flanked by 2 IS*26* elements) was on the chromosome of the epidemic strain and the IncHI1 plasmid but was not collinear, suggesting secondary rearrangements in the chromosomal AMR region induced by IS*26* elements.

**Figure 3:**
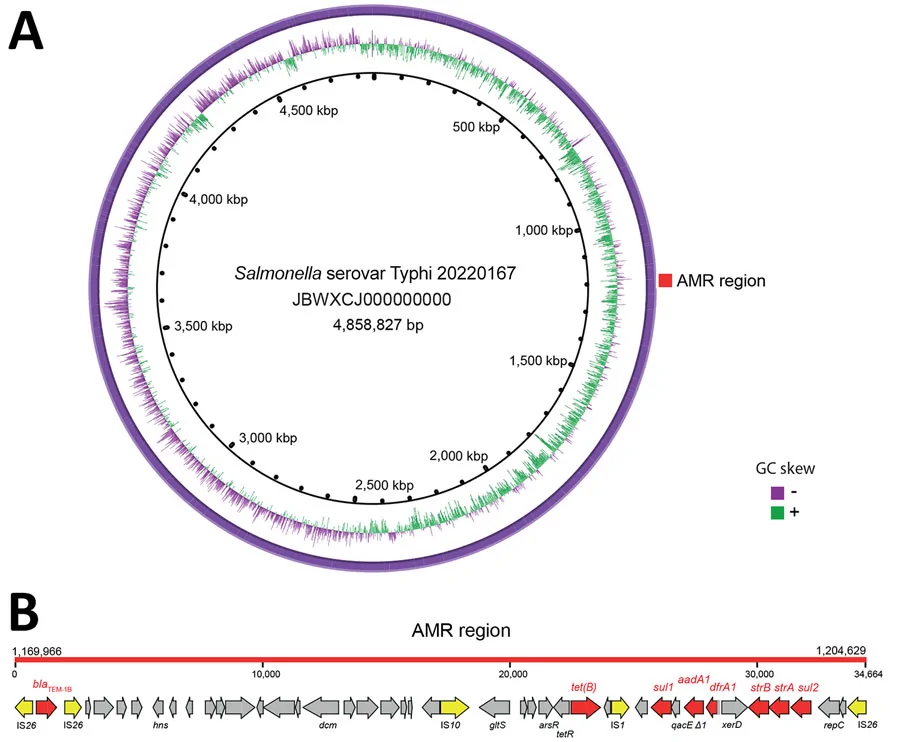
Chromosomal integration of an AMR region derived from an IncHI1 plasmid in study of emergence of genotype 2.3.1 multidrug-resistant *Salmonella enterica* serovar Typhi strain, West Africa, 2021–2025. A) Circular representation of the complete genome of isolate 202210167, a representative isolate of the emerging strain. Red square indicates the chromosomally integrated 34,664-bp AMR region in the external ring. B) Linear representation of the integrated AMR region containing the 8 AMR genes identified in the emerging strain (red indicates AMR genes, yellow indicates insertion sequences, and gray indicates other genes). AMR, antimicrobial resistance.

## Conclusion

We identified an emerging genotype 2.3.1 MDR *Salmonella* Typhi lineage in patients returning from sub-Saharan Africa, particularly Senegal. This lineage differs from the globally distributed MDR H58 (4.3.1 and derived) lineage originating from South Asia. Genomic analyses revealed the lineage’s evolution, including AMR acquisition. First identified in Cameroon in the 1950s, it acquired 2 distinct MDR plasmids during the 2000s. The IncHI1 plasmid was subsequentially lost, but its AMR region is now stably integrated into the bacterial chromosome. Since 2019, this lineage has acquired ciprofloxacin resistance through a chromosomal *gyrA* mutation. That trajectory closely mirrors that of H58, underscoring risk for further resistance development. Continued genomic surveillance is essential to monitor spread and guide control measures to prevent further resistance.

Appendix 1 Additional information about methods used in study of emergence of genotype 2.3.1 multidrug-resistant *Salmonella enterica* serovar Typhi strain, West Africa, 2021–2025.

Appendix 2 Additional information about individual isolates identified in study of emergence of genotype 2.3.1 multidrug-resistant *Salmonella enterica* serovar Typhi strain, West Africa, 2021–2025.
