# Supplementary material for: Emergence of Genotype 2.3.1 Multidrug-Resistant Salmonella enterica Serovar Typhi Strain, West Africa, 2021–2025
Source: Emerg Infect Dis. 2026 Oct;32(10):1736–40. doi: 10.3201/eid3210.260813 (PMC13632843; doi:10.3201/eid3210.260813)
Supplement: Supplementary file 1 [file 26-0813-Techapp-s1.pdf]

Article DOI: https://doi.org/10.3201/eid3210.260813

EID cannot ensure accessibility for supplementary materials supplied by authors. Readers who have difficulty accessing supplementary content should contact the authors for assistance.

Emergence of Genotype 2.3.1 Multidrug-Resistant *Salmonella enterica* Serovar Typhi Strain, West Africa, 2021–2025

Appendix 1

Serotyping

All isolates received by the French National Reference Center for *Escherichia coli*, *Shigella*, and *Salmonella* (FNRC-ESS) at Institut Pasteur, were confirmed to be *Salmonella* Typhi by conventional serotyping (*1*) until February 2019. After this date, typing was performed exclusively by whole-genome sequencing (see below).

Antimicrobial susceptibility testing

Until 31 December 2023, antimicrobial drug susceptibility testing (AST) was performed by disk diffusion on Mueller-Hinton (MH) agar (*2*). The following antimicrobial drugs (Bio-Rad, Marnes-la-Coquette, France) were tested: amoxicillin, ceftriaxone, ceftazidime, streptomycin, kanamycin, amikacin, gentamicin, nalidixic acid, ofloxacin, ciprofloxacin, sulfonamides, trimethoprim, sulfamethoxazole-trimethoprim, chloramphenicol, tetracycline, and azithromycin. The minimal inhibitory concentrations (MICs) of nalidixic acid and ciprofloxacin were determined with Etest strips (bioMérieux, Marcy L'Etoile, France).

From January 1, 2024, AST was performed by the microdilution method with EUVSEC3 plates (Sensititre, Thermo Fisher Scientific, Cleveland, OH, USA). *Escherichia coli* CIP 76.24 (ATCC 25922) was used as a control. AST results were interpreted in accordance with the guidelines of the Antibiogram Committee of the French Society for Microbiology (CA-SFM) / European Committee on Antimicrobial Susceptibility Testing (EUCAST) (*2*). The clinical breakpoint for ciprofloxacin susceptibility in isolates of *Salmonella* spp. is identical between

CA-SFM / EUCAST and Clinical & Laboratory Standards Institute (CLSI) guidelines, at an MIC ≤0.06 mg/L (*2*,*3*). However, the clinical breakpoint defining ciprofloxacin resistance in isolates of this species differs between guidelines, with resistance when MIC >0.06 mg/L for CA-SFM / EUCAST (*2*) and when MIC ≥1 mg/L (intermediate resistance, 0.12–0.5 mg/L) for CLSI (*3*).

Isolates were considered to be multidrug-resistant (MDR) if they were resistant to ≥3 of the following antimicrobial drugs: amoxicillin, cotrimoxazole (or after 2023 to both trimethoprim and sulfamethoxazole), chloramphenicol, or tetracyclines.

Short-read sequencing

Genomic typing has been used as the first-line typing method since February 2019. A retrospective sequencing of isolates received between January 2016 and January 2019 was also performed. Our genomic dataset therefore consists of 1,603 *Salmonella* Typhi isolates, 958 from mainland France and 645 from French overseas territories.

Short-read sequencing was performed as a routine procedure at the FNRC-ESS, and at the Mutualized Platform for Microbiology (P2M) at Institut Pasteur, Paris. Total DNA was extracted with the MagNA Pure DNA isolation kit (Roche Molecular Systems, Indianapolis, IN, USA). Sequencing libraries were prepared with the Nextera XT kit (Illumina, San Diego, CA, USA), and sequencing was performed with the NextSeq 500 system (Illumina). All reads were filtered with FqCleanER v.23.07 (https://gitlab.pasteur.fr/GIPhy/fqCleanER) to eliminate adaptor sequences and discard low-quality reads with phred scores <28 and a length <70 bp. Assemblies were generated with SPAdes v.3.15.5, with the following options: k-values of 21, 29, 37, 45, 53, 61, 69, and 77, and default settings for other criteria (*4*,*5*). Short-read sequence data were uploaded onto EnteroBase (https://enterobase.warwick.ac.uk) (*6*).

Genomic sequence analyses

Serovar Typhi prediction was based on the *in silico* serovar prediction tool SISTR1 (*7*) and/or clustering within multilocus sequence typing (MLST) eBurst group eBG13 (*8*) and core-genome MLST (cgMLST) hierarchical cluster (HC) HC900_202 (*9*); all these tools are integrated into EnteroBase (https://enterobase.warwick.ac.uk/species/index/senterica).

Genotypes were assigned with the *Salmonella* Typhi genotyping framework implemented in Mykrobe (v.0.13.0) on Illumina reads (*10*,*11*).

The presence and type of antimicrobial resistance genes — acquired or chromosomal point mutations — were determined with ResFinder v.4.5.0 with default parameters (http://genepi.food.dtu.dk/resfinder) (*12*) on SPAdes assemblies. The presence and type of plasmid replicons were determined with PlasmidFinder v.2.1 (https://cge.food.dtu.dk/services/PlasmidFinder-2.0) (*13*) on SPAdes assemblies.

Additional genomic data

Raw sequence files for the genomes of 40 published genotype 2.3.1 *Salmonella* Typhi isolates were downloaded from the ENA or GenBank and included in this study (Appendix 2, https://wwwnc.cdc.gov/EID/article/32/10/26-0813-App2.xlsx).

Phylogenetic analysis

Phylogenetic analyses were carried out on 207 genotype 2.3.1 *Salmonella* Typhi genomes, including 40 additional genomes retrieved from Carey *et al.* (*14*) (Appendix 2) Briefly, a core-genome single-nucleotide variant (SNV) alignment was generated with snippy v. 4.6.0 https://github.com/tseemann/snippy), by mapping paired-end filtered reads onto the reference genome of *Salmonella* Typhi CT18 (genotype 3.2.1, GenBank accession number AL513382), with the following parameters for a variant to be reported: mapping quality of 60, minimum base quality of 13, minimum read coverage of 4 and 75% read concordance. Repeats and phage regions (https://github.com/katholt/typhoid/blob/master/CT18_final_repeats_phage_forParseSNPTable.csv) were masked from the core-genome SNV alignment, and putative recombinogenic regions were detected with Gubbins v.3.2.0 (*15*). A maximum-likelihood (ML) phylogenetic tree was built from the alignment (1,130 SNVs) with RAxML v.8.2.12 (*16*), under the GTR model with 200 bootstraps, and visualized with iTOL v.7.5 (https://itol.embl.de) (*17*).

Bacterial conjugation assays

Resistance transfer experiments were performed on solid medium with *Escherichia coli* K-12 J5 resistant to sodium azide as the recipient strain. Two *Salmonella* Typhi isolates (IPCP and 201607514) were used as donor strains. Conjugation assays were performed by mixing equal volumes (100 µL) of 6-hour cultures of donor and recipient strains. The suspension was applied onto a 0.05 µm membrane filter (Merck Millipore, Burlington, MA, USA) on Luria Bertani (LB) agar (Difco, Becton Dickinson, Le Pont de Claix, France) and incubated at 37°C overnight. Cells were collected in 5 mL of sterile saline and were serially diluted before plating. Transconjugants were selected on Drigalski agar (Bio-Rad) supplemented with sodium azide (500 mg/L) and ampicillin (100 mg/L). Three *E. coli* transconjugants were arbitrarily selected in each experiment.

Long-read sequencing and complete genome circularization

For reconstruction of the full chromosomes and/or IncHI1 plasmids, four isolates of *Salmonella* Typhi genotype 2.3.1 (202210167, 202207569, 201607514, and IPCP) were selected and sequenced with Nanopore technology R10.4.1 (Oxford Nanopore Technologies, Oxford, United Kingdom) on the Plasmidsaurus sequencing platform (https://plasmidsaurus.com) after DNA extraction with the Genomic-tip 100/G kit (Qiagen, Hilden, Germany). We also sequenced two *E. coli* transconjugants (pIPCP-1 and p201607514–2). Draft and hybrid assemblies were obtained as previously described by Pulford *et al.* (*18*). Briefly, after sequencing, the genomic sequences of the isolates were assembled from long and short reads, with a hybrid approach, using Hybracter v.0.7.3 (https://github.com/gbouras13/hybracter) (*19*) for the *E. coli* transconjugants or Unicycler v.0.4 (https://github.com/rrwick/unicycler) (*20*) for the other three genomes.

Data availability

The list of the 167 genotype 2.3.1 *Salmonella* Typhi genomes studied (and their assembled short-read data) can be obtained from EnteroBase at: https://enterobase.warwick.ac.uk/species/senterica/search_strains?query = workspace:154089

Short-read sequence data were submitted to the European Nucleotide Archive (ENA) (http://www.ebi.ac.uk/ena), under study accession number PRJEB68323, with individual sequence accession numbers provided in Appendix 2.

The long-read sequence data generated in this study are available from GenBank (https://www.ncbi.nlm.nih.gov/genbank) under study accession number PRJNA1435116, with individual sequence accession numbers provided in Appendix 2.

References

1. Langendorf C, Le Hello S, Moumouni A, Gouali M, Mamaty AA, Grais RF, et al. Enteric bacterial pathogens in children with diarrhea in Niger: diversity and antimicrobial resistance. PLoS One. 2015;10:e0120275. PubMed https://doi.org/10.1371/journal.pone.0120275

2. Comité de l'Antibiogramme de la Société Française de Microbiologie (CA-SFM) and the European Committee on Antimicrobial Susceptibility Testing (EUCAST). Recommendations 2025. Volume 1.1 [in French]. 2025 Jul [cited 2026 Apr 24]. https://www.sfm-microbiologie.org/wp-content/uploads/2025/07/CASFM2025_V1.1-JUILLET-2025.pdf

3. Clinical and Laboratory Standards Institute. Performance standards for antimicrobial susceptibility testing. M100. Wayne (PA): The Institute; 2026.

4. Prjibelski A, Antipov D, Meleshko D, Lapidus A, Korobeynikov A. Using SPAdes De Novo Assembler. Curr Protoc Bioinformatics. 2020;70:e102. PubMed https://doi.org/10.1002/cpbi.102

5. Bankevich A, Nurk S, Antipov D, Gurevich AA, Dvorkin M, Kulikov AS, et al. SPAdes: a new genome assembly algorithm and its applications to single-cell sequencing. J Comput Biol. 2012;19:455–77. PubMed https://doi.org/10.1089/cmb.2012.0021

6. Zhou Z, Alikhan NF, Mohamed K, Fan Y, Achtman M; Agama Study Group. The EnteroBase user's guide, with case studies on *Salmonella* transmissions, *Yersinia pestis* phylogeny, and *Escherichia* core genomic diversity. Genome Res. 2020;30:138–52. PubMed https://doi.org/10.1101/gr.251678.119

7. Yoshida CE, Kruczkiewicz P, Laing CR, Lingohr EJ, Gannon VPJ, Nash JHE, et al. The *Salmonella* in Silico Typing Resource (SISTR): an open web-accessible tool for rapidly typing and subtyping draft *Salmonella* genome assemblies. PLoS One. 2016;11:e0147101. PubMed https://doi.org/10.1371/journal.pone.0147101

8. Achtman M, Wain J, Weill FX, Nair S, Zhou Z, Sangal V, et al.; *S. enterica* MLST Study Group. Multilocus sequence typing as a replacement for serotyping in *Salmonella enterica*. PLoS Pathog. 2012;8:e1002776. PubMed https://doi.org/10.1371/journal.ppat.1002776

9. Achtman M, Zhou Z, Charlesworth J, Baxter L. EnteroBase: hierarchical clustering of 100 000s of bacterial genomes into species/subspecies and populations. Philos Trans R Soc Lond B Biol Sci. 2022;377:20210240. PubMed https://doi.org/10.1098/rstb.2021.0240

10. Wong VK, Baker S, Connor TR, Pickard D, Page AJ, Dave J, et al.; International Typhoid Consortium. An extended genotyping framework for *Salmonella enterica* serovar Typhi, the cause of human typhoid. Nat Commun. 2016;7:12827. PubMed https://doi.org/10.1038/ncomms12827

11. Ingle DJ, Hawkey J, Hunt M, Iqbal Z, Keane JA, Afolayan AO, et al.; Global Typhoid Genomics Consortium. Typhi Mykrobe: fast and accurate lineage identification and antimicrobial resistance genotyping directly from sequence reads for the typhoid fever agent *Salmonella* Typhi. Genome Med. 2025;17:130. PubMed https://doi.org/10.1186/s13073-025-01551-4

12. Florensa AF, Kaas RS, Clausen PTLC, Aytan-Aktug D, Aarestrup FM. ResFinder—an open online resource for identification of antimicrobial resistance genes in next-generation sequencing data and prediction of phenotypes from genotypes. Microb Genom. 2022;8:000748. PubMed https://doi.org/10.1099/mgen.0.000748

13. Carattoli A, Zankari E, García-Fernández A, Voldby Larsen M, Lund O, Villa L, et al. In silico detection and typing of plasmids using PlasmidFinder and plasmid multilocus sequence typing. Antimicrob Agents Chemother. 2014;58:3895–903. PubMed https://doi.org/10.1128/AAC.02412-14

14. Carey ME, Dyson ZA, Ingle DJ, Amir A, Aworh MK, Chattaway MA, et al.; Global Typhoid Genomics Consortium. Global diversity and antimicrobial resistance of typhoid fever pathogens: Insights from a meta-analysis of 13,000 *Salmonella* Typhi genomes. eLife. 2023;12:e85867. PubMed https://doi.org/10.7554/eLife.85867

15. Croucher NJ, Page AJ, Connor TR, Delaney AJ, Keane JA, Bentley SD, et al. Rapid phylogenetic analysis of large samples of recombinant bacterial whole genome sequences using Gubbins. Nucleic Acids Res. 2015;43:e15–15. PubMed https://doi.org/10.1093/nar/gku1196

16. Stamatakis A. RAxML version 8: a tool for phylogenetic analysis and post-analysis of large phylogenies. Bioinformatics. 2014;30:1312–3. PubMed https://doi.org/10.1093/bioinformatics/btu033

17. Letunic I, Bork P. Interactive Tree of Life (iTOL) v6: recent updates to the phylogenetic tree display and annotation tool. Nucleic Acids Res. 2024;52(W1):W78–82. PubMed https://doi.org/10.1093/nar/gkae268

18. Pulford CV, Perez-Sepulveda BM, Ingle DJ, Bengtsson RJ, Bennett RJ, Rodwell EV, et al. Global diversity and evolution of *Salmonella enterica* serovar Panama: a genomic epidemiology study. Lancet Microbe. 2025;6:101150. PubMed https://doi.org/10.1016/j.lanmic.2025.101150

19. Bouras G, Houtak G, Wick RR, Mallawaarachchi V, Roach MJ, Papudeshi B, et al. Hybracter: enabling scalable, automated, complete and accurate bacterial genome assemblies. Microb Genom. 2024;10:001244. PubMed https://doi.org/10.1099/mgen.0.001244

20. Wick RR, Judd LM, Gorrie CL, Holt KE. Unicycler: resolving bacterial genome assemblies from short and long sequencing reads. PLOS Comput Biol. 2017;13:e1005595. PubMed https://doi.org/10.1371/journal.pcbi.1005595
